# Improved conductive carbon nanotube tape using natural cellulose fibrils for array tomography

**DOI:** 10.1186/s42649-026-00134-w

**Published:** 2026-05-09

**Authors:** Minkyo Jung, Hyosun Choi, Chang Kee Lee, Ji Young Mun

**Affiliations:** 1 Neural Circuit Research Group, Korea Brain Research Institute, Daegu, Korea; 2 Korea Institute of Industrial Technology, Ansan, Korea; 3 National Instrumentation Center for Environmental Management, Seoul National University, Seoul, Korea

**Keywords:** Automated tape-collecting ultramicrotomy, Carbon nanotube, Cellulose, Hydrophilic, Conductivity

## Abstract

Array tomography using scanning electron microscopy (SEM) enables large-volume, nanometer-scale reconstruction of neural circuits and allows nanometer resolution of synaptic vesicles and other ultrastructural features. For stable serial imaging, Kapton tape, carbon nanotube (CNT) tape mounted on silicon wafers, and indium tin oxide (ITO) glass have commonly been used. However, each tape shows practical limitations: CNT tape is not consistently available, whereas Kapton tape often requires additional glow discharge or carbon coating steps to reduce charging artifacts and improve imaging stability.

Here, we report the development of a lab-made tape using a cellulose/CNT/AgNW composite tape designed for SEM-based array tomography. By integrating intrinsically hydrophilic natural cellulose fibrils with conductive carbon or silver nanotubes, the newly developed tape provides sufficient surface hydrophilicity and conductivity without additional glow discharge or carbon coating treatment. This simplifies workflow while maintaining stable imaging conditions. Notably, the cellulose fibrils containing tape demonstrated reduced charging artifacts, improved section adhesion, and stable high-resolution imaging suitable for nanometer level analysis. Our results establish cellulose-based conductive tape as a practical advancement for array tomography, enhancing reliability and reproducibility in large-scale SEM imaging.

## Introduction

Scanning electron microscopy (SEM) provides significant advantages for large area imaging to study cellular interactions at nanometer resolution. Especially, three-dimensional volume electron microscopy including serial block face (SBF)-scanning electron microscopy (SEM) and focused ion beam SEM (FIB-SEM) have become widely used techniques in neuroscience. These approaches rely on in situ destructive sectioning within the SEM vacuum chamber, either by a diamond knife or by a focused ion beam milling (FIB-SEM) (Kubota et al. 2018a; Denk and Horstmann 2004). When in situ destructive sectioning approaches including SBF-SEM and FIB-SEM are not utilized, preparing hundreds or even thousands of serial thin sections of biological samples remain technically demanding and often requires substantial operator expertise.

Moreover, sections collected on fragile formvar film–coated single-slot EM grids are easy to break the supporting formvar film, which can interrupt serial imaging for 3D volume electron microscopy. While these methods reduce the need for manual serial section collection and alignment, their field of view is typically limited to approximately 0.1 × 0.1 mm^2^ and 0.5 × 0.5 mm^2^ (Wanner et al. 2015; Hua et al. 2015; Wilke et al. 2013). To overcome these limitations, automatic tape-collecting ultramicrotomy (ATUM) has emerged as an advanced approach for large-volume SEM imaging (Hua et al. 2015). ATUM enables automated collection of thousands of serial ultrathin sections onto tape for storage. Combined with EM navigation and mosaic acquisition software such as MAPS or ATLAS 5, millimeter-scale volumes can be imaged serially (Hayworth et al. 2014).

For stable imaging, another essential material is tape for ATUM (Kubota et al. 2018b). Currently, Kapton tape is the most widely available commercial material. Although Kapton tape offers stable supply and mechanical robustness, it exhibits limited electrical conductivity and insufficient surface hydrophilicity. Carbon nanotube (CNT)-coated polyethylene terephthalate (PET) tape was reported in 2018 as an improved surface conductivity and enhanced imaging stability. Despite its technical advantages, glow-discharged CNT–PET tape is not commercially available. To address these limitations, we developed a composite tape comprising natural cellulose fibrils, carbon nanotubes, and silver nanowires deposited on a PET substrate. By integrating intrinsically hydrophilic natural cellulose fibrils with conductive CNTs through a simple and eco-friendly mechanical dispersion (Wentao et al. 2019; Choi et al. 2017; Hwang et al. 2018), we aimed to achieve both stable surface hydrophilicity and sufficient conductivity without requiring glow discharge or additional carbon coating. Properties such as conductivity, hydrophilicity, resistance to beam damage, mechanical and chemical strength, and surface structure are critical for ATUM tape (Kubota et al. 2018b). Among these, the hydrophilicity and surface roughness of the substrate are key factors that determine the adhesion of the specimen, the flatness of the section, and the quality of high-resolution imaging. Given that we are dealing with ultra-thin sections tens of nanometers (nm) thick, a rough substrate has a critical impact on data alignment and resolution. In this study, we evaluated whether this composite tape could serve as a practical material for ATUM-based array tomography, while simplifying the workflow.

## Materials and methods

### Preparation of nanofiber, nanotube deposited tape and evaluation

Natural cellulose fibrils (CNF), carbon nanotubes (CNT), and silver nanowires (AgNWs) were used to fabricate conductive composite tapes. To ensure deagglomeration and uniform dispersion without chemical additives, the aqueous mixture of CNF and CNT was subjected to a high-power mechanical shaking process using zirconia beads (Park et al. 2024). This straightforward collision process enables the self-alignment of individual CNTs within the cellulose network, creating a homogeneous viscous dope. For the AgNW-integrated tape, AgNWs were subsequently introduced into the dispersion. Surface hydrophilicity of Kapton, CNT, CNF–CNT, and CNF–CNT–AgNW tapes was evaluated by static water contact angle (WCA) measurement. Electrical conductivity was measured using a standard four-point probe method to minimize contact resistance effects. To evaluate multi-scale surface roughness, measurements were conducted at two magnifications (× 200 and × 500), corresponding to two different analysis scales.

### EM Block preparation, ultrathin-sectioning, and imaging

Brain tissue was treated with intensive osmium solution and en bloc heavy metal staining. Those fixative and staining solutions helped to get high membrane contrast and avoid severe charging (Hua et al. 2015; Kim et al. 2020). Male mouse (*n* = 2) was deeply anesthetized and intracardially perfused with 2% paraformaldehyde and 2.5% glutaraldehyde in 0.15 M cacodylate buffer (pH 7.4). Brain slices (150 μm-thick) were made with a vibratome in ice-cold 0.15 M cacodylate buffer, and small pieces of hippocampal CA1 stratum radiatum were incubated in the same fixative at 4 ºC. After washing, samples were placed in cacodylate buffer containing 2% OsO4 and 1.5% potassium ferrocyanide for 1 h. Tissues were placed in 1% thiocarbohydrazide (TCH, Ted Pella, USA) solution for 20 min and then placed in 2% aqueous OsO_4_ for 30 min (OTO). Thereafter, tissues were incubated in 1% uranyl acetate at overnight and lead aspartate solution at for 30 min to enhance membrane contrast as described previously. The tissues were dehydrated using a graded series of acetone (50%, 70%, 80%, 90%, 95%, and 100%), infiltrated with mixture of resin and acetone, and 100% resin. The resin was prepared from the Epon 812 kit (EMS, USA). 50 nm thick ultrathin sections through Automatic tape ultramicrotomy (ATUM, RMC, USA) were done with different tapes. Sections were loaded on Kapton, carbon nanotube tape (CNT), and improved two kinds of carbon tape (CNF–CNT and CNF–CNT–AgNW). Each tape was attached to the wafer or SEM stub. To compare hydrophilicity for loading serial sections and conductivity, tapes were used glow discharge and carbon coating. Imaging was performed using a Gemini 300 SEM (Carl Zeiss Microscopy) equipped with a backscattered electron detector (BSD) at 5-kV beam voltage and a 7 μs dwell time.

## Results and discussion

Kapton (polyimide) tape is limited with wrinkled section due to contact angle and charging without glow discharging and carbon coating. Kubota et al. showed plasma-hydrophilized CNT coated PET tape to enhance imaging without wrinkling and charging. Plasma treatment and carbon coating solved the problem in their report (Kubota et al. 2018b). However, the CNT–PET tape described by Kubota et al. is unstable supply for routine laboratory use. Therefore, we developed CNT-PET tape and improved with natural cellulose fibrils and silver nanowires (AgNW) to enhance hydrophilicity and reduce charging effects. Because natural cellulose fibrils (CNF) possess sufficient hydrophilicity (Park et al. 2024), the tape does not require plasma treatment. In addition, AgNW can enhance conductivity to reduce charging effects without carbon coating.

**Fig. 1:**
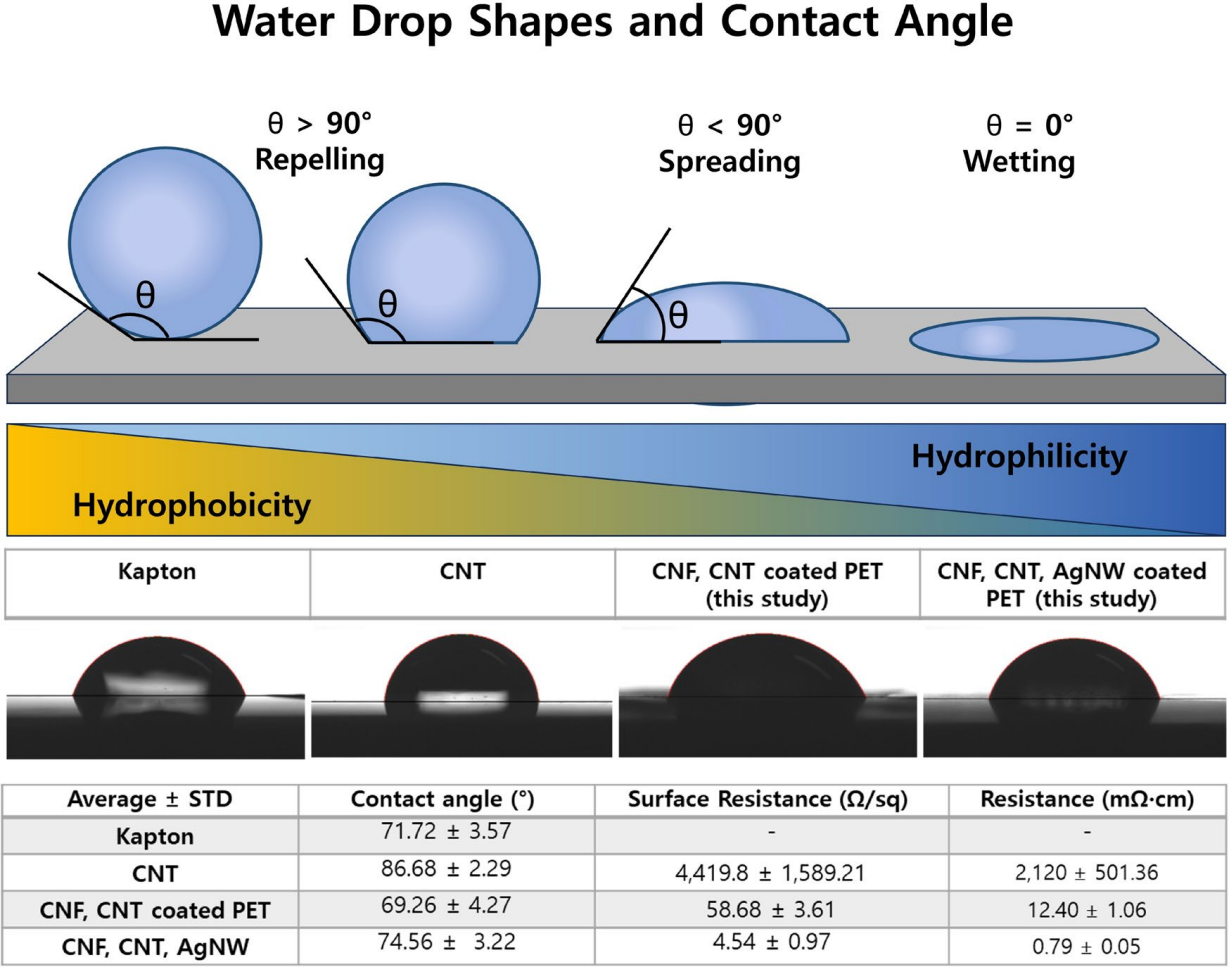
The illustration of water drop shapes and contact angle values was modified from (Krasowska et al. 2009), and is used with permission from Elsevier. Water contact angle (WCA) analysis of Kapton, CNT, CNF-CNT and CNF-CNT-AgNW coated PET tape. All tapes were used without glow discharge or carbon coating treatment. Representative side-view images of 3 μL deionized water droplets deposited on each tape are shown at the top. The red curve indicates the fitted droplet profile used for contact angle calculation. Measurements were performed at three different positions per sample, and the results are presented as mean ± standard deviation. Kapton exhibited a contact angle of 71.72 ± 3.57°, indicating moderate hydrophilicity. The CNF/CNT-coated PET surface showed a contact angle of 69.26 ± 4.27°, suggesting slightly increased hydrophilicity compared to Kapton. In addition, sheet resistance of the CNF/CNT-coated PET were measured, yielding values of 58.68 ± 3.61 and 12.40 ± 1.06 (mean ± SD), respectively. The combined hydrophilicity and roughness results indicate that the CNF/CNT coating modifies the surface morphology and slightly enhances surface hydrophilicity relative to Kapton. CNT vs CNF-CNT: *p* < 0.001. Data are presented as mean ± SD (*n* = 5). Statistical significance was assessed using a two-tailed Welch’s t-test

Water contact angle analysis demonstrated that CNF incorporation reduced the intrinsic hydrophobicity of CNT-PET tapes without plasma treatment. Although CNT-PET tape exhibited the highest WCA (86.68 ± 2.29°), the introduction of CNF reduced the angle to 69.26 ± 4.27°, comparable to Kapton (71.72 ± 3.57°) but without requiring glow discharge. CNF-CNT-AgNW maintained moderate hydrophilicity (74.56 ± 3.22°), indicating that AgNW incorporation did not significantly compromise surface wettability (Fig. 1). In ultra-sectioning, CNF-containing tapes showed uniform section with spreading. These findings suggest that contact angle below ~ 75° may be sufficient for stable section adhesion without plasma treatment.

Surface sheet resistance measurements confirmed that the CNF-CNT and CNF-CNT-AgNW tapes provide dramatically improved conductivity compared to Kapton. While the surface sheet resistance of Kapton was too high to be measured (exceeding the instrument’s limit), the values for CNT, CNF-CNT, and CNF-CNT-AgNW were 4,419 ± 1,589, 58.68 ± 3.61, and 4.54 ± 0.97 Ω/sq, respectively (Fig. 1). This remarkable enhancement in conductivity without chemical treatments or carbon coating can be attributed to the continuous collision process using zirconia beads. This process effectively separates bundled CNTs into individual nanotubes and self-aligns them along the cellulose fibril chains (Wentao et al. 2019), creating highly conductive networks. This conductivity improvement correlates directly with the suppression of charging artifacts during SEM imaging.

**Fig. 2:**
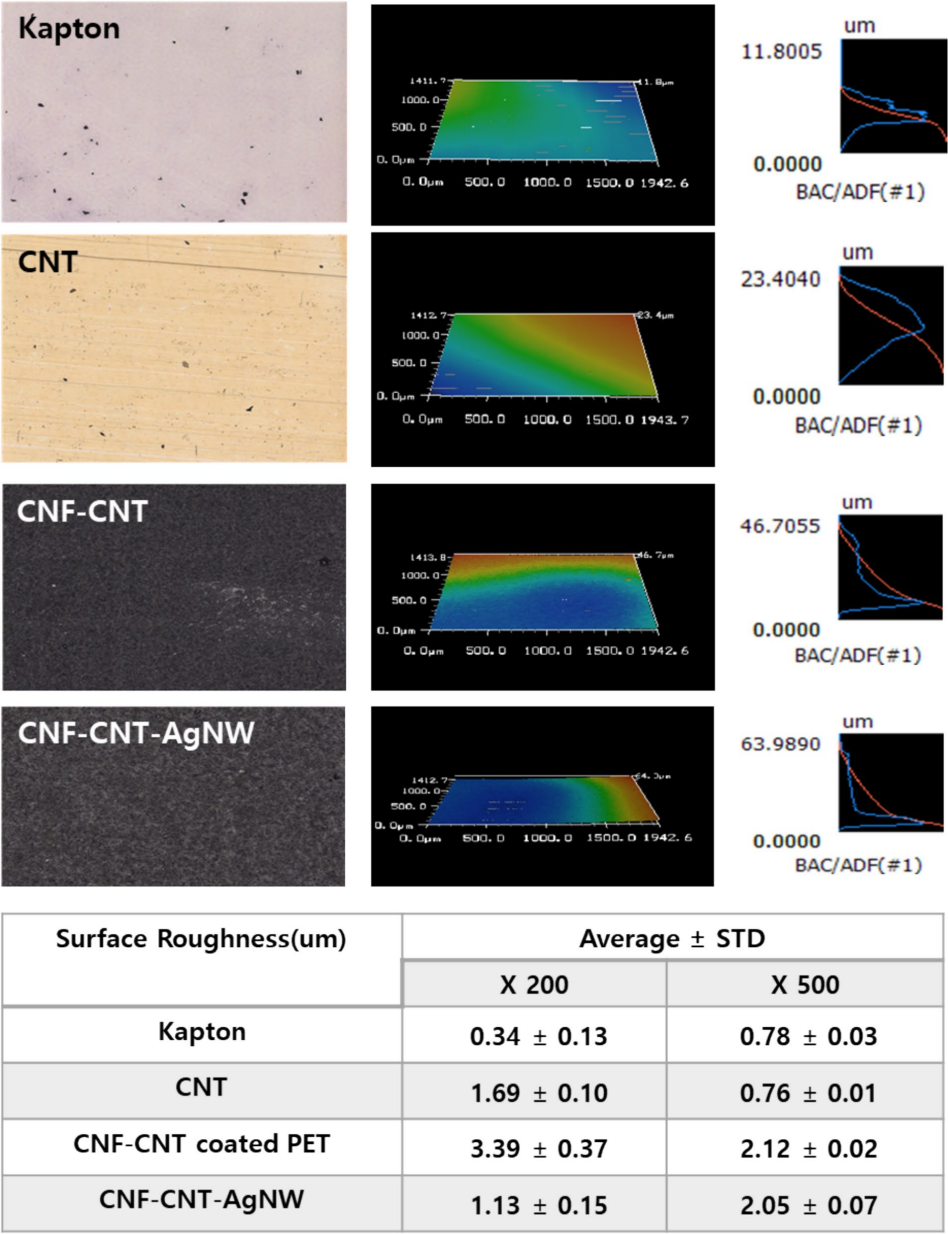
Surface morphology and roughness characterization of Kapton, CNT, CNF–CNT, and CNF–CNT–AgNW tapes. Representative optical microscopy images (left), 3D surface profilometry maps (middle), and corresponding height distribution profiles (right) of four different tape substrates: Kapton, CNT tape, CNF–CNT composite tape, and CNF–CNT–AgNW composite tape. Kapton and CNT tapes exhibit relatively smooth but directionally biased surfaces, with CNT tapes showing linear alignment features likely originating from the fabrication process. In contrast, CNF–CNT and CNF–CNT–AgNW tapes display more homogeneous and textured surface morphologies, reflecting the fibrous CNF network and embedded conductive nanomaterials. Roughness increased markedly upon CNF incorporation, while the effect of AgNW addition depended on magnification. The maximum height variations (µm scale) are indicated in each panel. Quantitative roughness analysis at × 200 and × 500 magnifications (bottom table) shows that CNF–CNT–AgNW tapes exhibit enhanced surface roughness compared to CNT tapes, particularly at higher magnification, indicating increased nanoscale surface heterogeneity. Data are presented as mean ± standard deviation. Profilometry maps (left) visualize the surface topography, and height distribution profiles (right) quantify the spread of surface heights (RMS roughness)

Surface roughness measurements revealed that CNF-CNT and CNF-CNT-AgNW tape exhibit higher roughness at low magnification (× 200) compared to Kapton and CNT. However, the roughness values of CNF-CNT and CNF-CNT-AgNW became similar to each other, although both remained higher than those of Kapton and CNT tape at × 500. These surface features may contribute to section adhesion and imaging behavior; however, the observed charging suppression is more directly associated with improved conductivity. The surface roughness was each 0.34 ± 0.13, 0.16 ± 0.10, 3.39 ± 0.37, and 1.13 ± 0.15 at × 200. In × 500, it was each 0.78 ± 0.03, 0.76 ± 0.01, 2.12 ± 0.02, and 2.05 ± 0.07 (Fig. 2). In Fig. 2, the optical images provide a qualitative overview of the surface appearance of each tape, whereas the 3D profilometry maps visualize the spatial distribution of surface height across the measured area. The corresponding height distribution profiles help compare the amplitude and uniformity of surface-height fluctuations among the samples. Together, these panels show that CNF-containing tapes have a more textured and heterogeneous surface than Kapton or CNT tape, consistent with the formation of a fibrous CNF-based network.

**Fig. 3:**
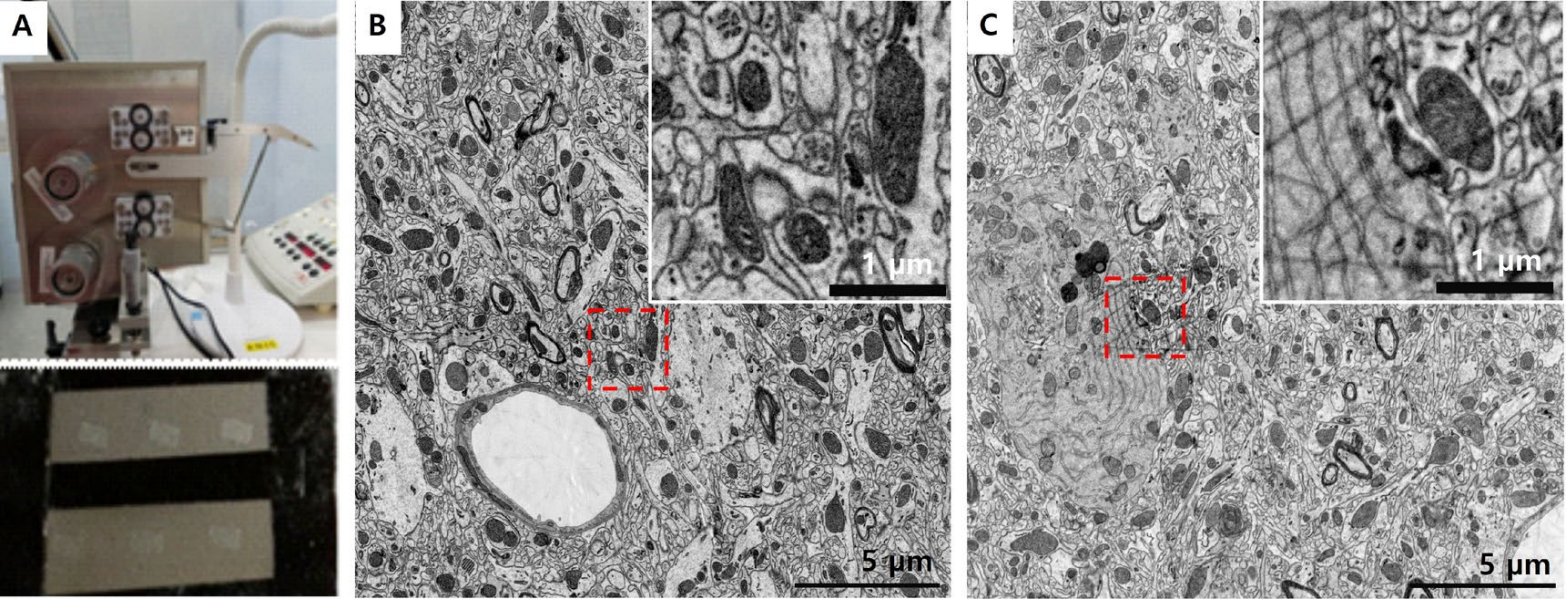
Application of the CNF–CNT tape in ATUM and SEM imaging without glow discharging and carbon coating. **A** CNF–CNT tape mounted on an Automated Tape-Collecting Ultramicrotome (ATUM) system for serial section collection. The tape was stably installed and continuously transported during sectioning. **B** Representative scanning electron microscopy (SEM) images of ultrathin brain tissue sections collected on the CNF-CNT tape. High-resolution imaging was achieved at the cellular organelle level, clearly showing mitochondria, vesicles, and myelinated axons. Notably, imaging was performed without glow discharging or carbon coating, and no significant charging artifacts were observed. **C** Representative SEM images of ultrathin brain tissue sections collected on the CNF-CNT-AgNW tape. In some areas, fiber-like signals(NF) are observed within the red dashed box. PSD; postsynaptic density, MT; mitochondria, NF; nanofiber

To test the improvement of this tape, we embedded mouse brain tissue in epoxy resin using OTO method and obtained serial ultrathin sections of 50 nm thickness using ATUM techniques (Fig. 3a). Tissue sections involving an en bloc osmium-tetroxide –thiocarbohydrazide (TCH) staining procedure can produce adjust contrast without post-staining. Basically, hydrophilization by plasma treatment and carbon coating should be done for reduction of wrinkles and charging to use Kapton tape. To compare SEM image of same section on CNF-CNT and CNF-CNT-AgNW tape, sections of mouse brain tissue were imaged without plasma treatment and carbon coating. In both CNF-CNT and CNF-CNT-AgNW tape without plasma treatment and carbon coating, organelles such as synaptic vesicle, mitochondria, and endoplasmic reticulum in neuronal cells were observed without wrinkles and charging (Fig. 3B and C).

However, for the CNF-CNT-AgNW tape, fiber-like signals were observed in some areas against the sample background (Fig. 3C red dashed box). Because plasma-treated surfaces gradually lose hydrophilicity over time, plasma re-treatment is necessary for other tapes, which is inconvenient and time-consuming. In contrast, the intrinsic and stable hydrophilicity of CNF is a major advantage. In addition, conventional ATUM-SEM workflows using Kapton tape also require carbon coating to suppress charging. This step often makes obstacles due to variability in coating thickness and time consuming for serial EM. The AgNW-integrated CNF-CNT tape achieved substantially lower sheet resistance, enabling stable SEM imaging without coating. However, it is critical that the AgNW nanomaterials are uniformly and well-dispersed within the material. Otherwise, AgNW aggregates may appear in the acquired images and interfere with high-resolution imaging quality (Fig. 3C).

## Conclusions

CNF-CNT and CNF-CNT-AgNW tapes enable wrinkle-free, charging-free SEM imaging of ultrathin brain sections without plasma treatment or carbon coating. The material-level integration of hydrophilic nanofibers and conductive nanowires, created via a simple and chemical-free collision process, provides an alternative to conventional Kapton-based ATUM tape. By promoting the structural self-alignment of highly conductive networks within natural cellulose, this straightforward approach simplifies the workflow and significantly enhances ultrastructural imaging fidelity for large-scale serial SEM imaging.

## Data Availability

The datasets used and/or analyzed during the current study are available from the corresponding author on reasonable request.
